# Association Between Health Care Utilization and Immigration Enforcement Events in San Francisco

**DOI:** 10.1001/jamanetworkopen.2020.25065

**Published:** 2020-11-10

**Authors:** Vasil I. Yasenov, Jens Hainmueller, Michael Hotard, Duncan Lawrence, Laura M. Gottlieb, Jacqueline M. Torres

**Affiliations:** 1 Immigration Policy Lab, Stanford University, Stanford, California; 2 Institute of Labor Economics, Bonn, Germany; 3 Department of Political Science, Stanford University, Stanford, California; 4 Department of Family and Community Medicine, University of California, San Francisco; 5 Department of Epidemiology and Biostatistics, University of California, San Francisco

## Abstract

This cohort study explores whether an inclusive, local health care system could serve as a buffer against adverse effects on access to health care due to actions related to immigration status in patients who are likely undocumented.

## Introduction

Researchers have documented a negative association between immigration enforcement and health care utilization among immigrants^1^ and expressed concern about decreased utilization after the 2016 US presidential election.^2,3^ We explored whether an inclusive, local health care system in San Francisco acts as a buffer against adverse utilization effects of enforcement and related political events among patients who likely have undocumented immigration status.

## Methods

Data for this cohort study came from a single large, integrated health system that provides services to patient members of Healthy San Francisco (HSF), a health care program that provides access to a broad array of health care services to adults unable to access other public insurance options.^4^ San Francisco Health Network includes primary and specialty clinics and a hospital and trauma center and serves as the medical home for most patient members of HSF.^4^ We extracted all clinical encounter records in the San Francisco Health Network between November 1, 2015, and March 1, 2018 (168 975 encounters, 22 525 patients). This study was approved by the institutional review board of the University of California San Francisco. A waiver of informed consent was not required by the institutional review board. This study followed the Strengthening the Reporting of Observational Studies in Epidemiology (STROBE) reporting guidelines.

After California’s Medi-Cal expansion took effect, immigration status was the primary reason HSF members were ineligible for other types of insurance.^4^ Individuals with undocumented immigration status are generally excluded from public health insurance programs such as Medi-Cal. We used participation in HSF as a proxy for adults’ immigration status.^5^ For analyses of adults, the 2 groups we expected would be most affected were (1) all patients who had all encounters billed to HSF (HSF always) and (2) Hispanic patients who had at least 1 encounter billed to HSF between November 1, 2015, and March 1, 2018 (HSF ever, Hispanic). Groups we expected would be less affected or not affected were Hispanic patients and non-Hispanic patients who had encounters billed to Medi-Cal only (Medi-Cal always, Hispanic and Medi-Cal always, non-Hispanic). For analyses of pediatric patients, the group we expected to be more affected was Hispanic children and the group we expected to be less affected was non-Hispanic children.

**Figure 1.**
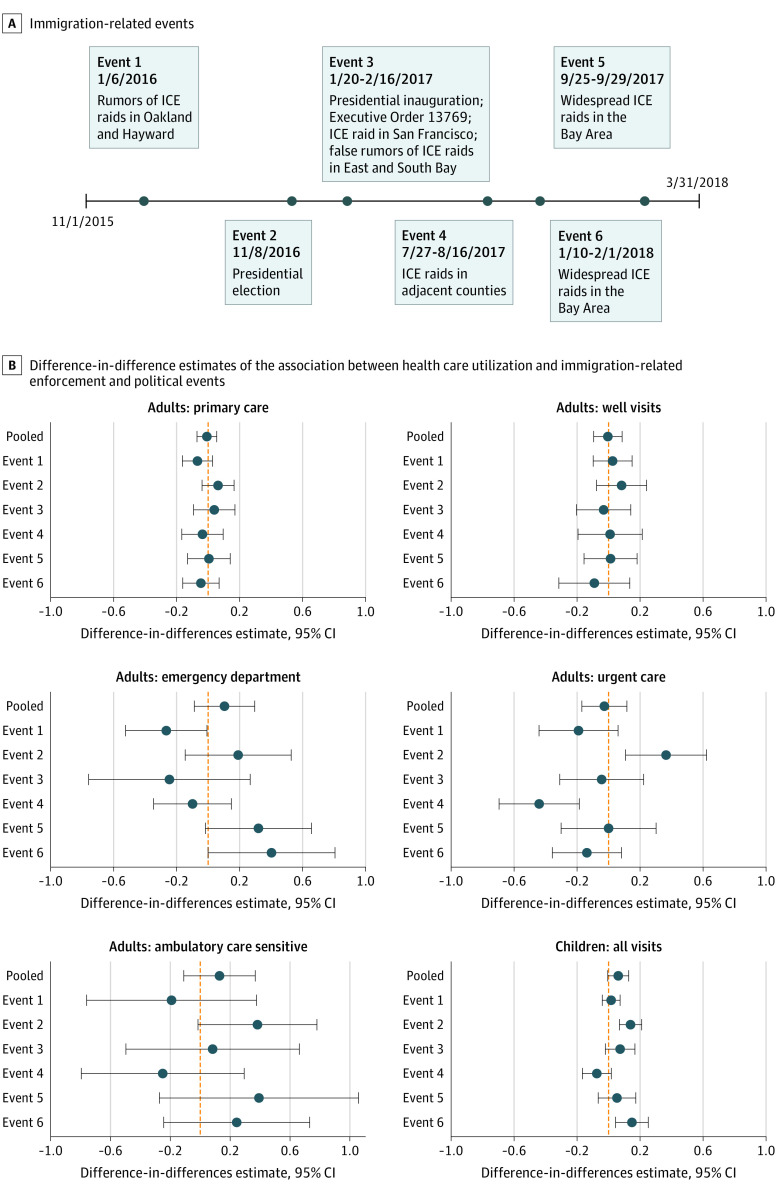
Immigration-Related Events and Association Between Health Care Utilization and Immigration Enforcement Events A, Timeline of immigration-related enforcement and policy events analyzed in this article. B, Difference-in-differences estimates of the association between these events and health care utilization among patients hypothesized to be most likely affected by these events. ICE indicates Immigration and Customs Enforcement.

We identified 6 periods in which actual or anticipated adverse immigration policy or enforcement events (eg, local Immigration and Customs Enforcement raids, immigration enforcement executive orders, the 2016 US presidential election) occurred at the federal or local level (Figure 1). The 3 primary outcomes were the log number of encounters in primary care clinics, urgent care, and emergency departments. We also examined preventive care visits in primary care clinics, emergency department encounters specific to ambulatory care–sensitive conditions, and pediatric patient visits across all health care settings.

Our analysis was at the week-group level covering 5 weeks before and after each event. We also pooled all 6 events and groups to achieve statistical precision. We used a difference-in-differences design controlling for week and group fixed effects. Stata Statistical Software (release 15.1) was used for analysis. Significance was set at *P* < .05, and tests were 2-sided. See the eAppendix in the Supplement for additional methodological details.

## Results

**Figure 2.**
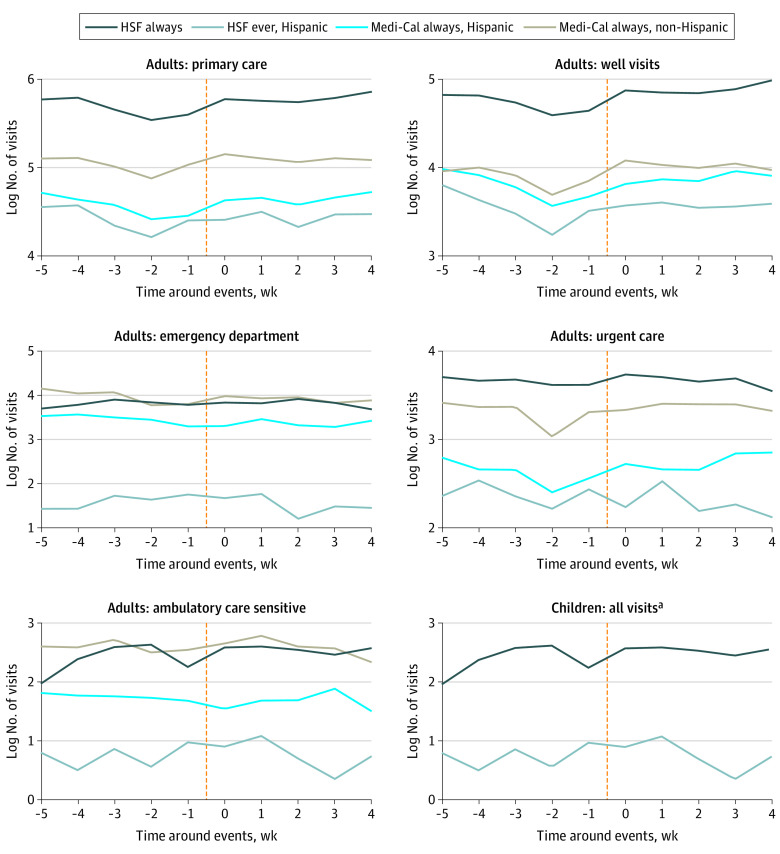
Trends in Health Care Utilization Before and After Immigration-Related Enforcement and Political Events (Pooled Analysis) Health care utilization in the 5 adult and 1 pediatric comparison groups. All estimates reflect pooled utilization for all 6 immigration-related events defined in Figure 1. HSF indicates Healthy San Francisco. ^a^The comparison groups for children are for Hispanic vs non-Hispanic patients.

Among the 168 975 encounters involving 22 525 patients, 2815 patients (12.5%) were included in the HSF always group; 4627 (20.5%) in the HSF ever, Hispanic group; 5001 (22.2%) in the Medi-Cal always, Hispanic group; and 10 082 (44.8%) in the Medi-Cal always, non-Hispanic group. Plots of pre-event health care utilization suggested parallel trends before each event across groups and settings (Figure 2). In pooled estimates that compared outcomes for groups likely to be most affected with outcomes for less affected groups across all events, there were no significant associations between immigration events and utilization of primary care (difference in differences estimate, −0.008; 95% CI, −0.07 to 0.05), urgent care (difference in differences estimate, −0.024; 95% CI, −0.17 to 0.12), or the emergency department (difference in differences estimate, 0.11; 95% CI, −0.08 to 0.30) (Figure 1).

## Discussion

Prior research has documented an association between decreased health care utilization and immigration enforcement in Alabama and Arizona.^1,2^ We did not find systematic evidence of an association between enforcement events and changes in utilization among patients with potentially undocumented immigration status in San Francisco. This suggests that a local environment with inclusive health care policies may mitigate the consequences of immigration enforcement actions. Study limitations include selection bias due to the clinical sample, the use of proxies for immigration status, and lack of power to evaluate specific reasons for clinical presentation (eg, acute stress). Future research should examine this question in a broader set of communities with varying degrees of health care policy inclusiveness and could more deeply explore the racialized nature of immigration enforcement and health consequences for Hispanic patients.^6^

## Notes

Supplement. **eAppendix.** Supplemental Methods **eReferences.**
