# Supplementary material for: Association Between Health Care Utilization and Immigration Enforcement Events in San Francisco
Source: JAMA Netw Open. 2020 Nov 10;3(11):e2025065. doi: 10.1001/jamanetworkopen.2020.25065 (PMC7656285; doi:10.1001/jamanetworkopen.2020.25065)
Supplement: Supplement. — eAppendix. Supplemental Methods eReferences. [file jamanetwopen-e2025065-s001.pdf]

Supplemental Online Content

Yasenov VI, Hainmueller J, Hotard M, Lawrence D, Gottlieb LM, Torres JM. Association between health care utilization and immigration enforcement events in San Francisco. *JAMA Netw Open*. 2020;3(11):e2025065. doi:10.1001/jamanetworkopen.2020.25065

eAppendix. Supplemental Methods

eReferences

This supplemental material has been provided by the authors to give readers additional information about their work.

© 2020 Yasenov VI et al. *JAMA Network Open.*

eAppendix. Supplemental Methods

1. Data

1.1 Source

Data for the analyses come from electronic medical records covering all visits in all healthcare facilities in the San Francisco Health Network (SFHN) from November 2015 until February 2018. SFHN is an integrated health care delivery system run by San Francisco Public Health Department (SFPHD) that includes primary and specialty clinics as well as the Zuckerberg San Francisco General (ZSFG) hospital and trauma center. Data were de-identified with the exception of date of service. The data include demographic information on all patients as well as the location, diagnoses and insurance codes for each visit. SFHN is the home for the majority of Healthy San Francisco (HSF) members. HSF is a health access program for residents of San Francisco that allows uninsured residents to access affordable health care services. In 2016-2017, 13,615 people were enrolled in the program.

1.2. Outcomes

The three primary outcomes considered were the log number of encounters for adult patients (18+) in each of the following settings: primary care clinics, urgent care clinics; the emergency department (ED). We also examined the log number of wellness or preventive visits in primary care clinics. The first three outcomes are defined based on the facility in which the encounter took place (e.g., an ED or an urgent care clinic) while the wellness or preventive visits are identified based on ICD-10 codes.[1]

In an additional analysis, we focused on emergency department visits for ambulatory care sensitive conditions among adult patients. These encounters were defined based on both hospital facility (i.e. ED) and ICD-10 codes as listed in Eggli et al. (2014).[2] These codes are designed to isolate ED encounters due to complications which could have been avoided under regular healthcare utilization. These could arise if, for instance, patients initially avoided seeking healthcare as a result of immigration-related events, contributing to the worsening of underlying medical conditions.

1.3. Sample

The sample for the primary analyses consisted of all patients age 18 and over who appeared in the data at least once in the year prior to the 2016 US Presidential election (i.e. November 2015 – November 2016). This second restriction limited the potential for spurious results driven by

[1] General and other special examinations without complaint and encounters for immunization.

[2] The list is based in part on original article by Purdy et al. (2009). These included angina, asthma, bacterial pneumonia, cellulitis, congestive heart failure, chronic obstructive pulmonary disease, dehydration and gastroenteritis, infectious/food-borne gastroenteritis, dental conditions, diabetes complications, ear, nose and throat infections, hypertension, hypoglycemia, influenza and pneumonia, iron or other nutritional deficiency anemia, nutritional deficiency, other vaccine preventable diseases, pelvic inflammatory disease, perforated/bleeding ulcer, pyelonephritis, appendicitis with rupture.

© 2020 Yasenov VI et al. *JAMA Network Open.*

compositional changes in the San Francisco population resulting from the outcome of the election. For instance, it is possible that more immigrant patients moved to or away from San Francisco or to non-SFHN health care settings within San Francisco post-election which will result in either higher or lower observed healthcare utilization among this subgroup. This is a different effect than the one we aimed to isolate. However, we conducted additional tests to evaluate potential selection bias generated by this sample restriction and examined all new patients to determine whether our null results could be driven by compositional changes of HSF and Medi-Cal patients (aggregated across all four types of visits to the week-group level). In 2016, Latinx patients accounted for close to 75 percent. In the final set of analyses, we focus separately on children age 17 and younger who appeared in the data at least once in the year prior to the 2016 US Presidential election.

1.4. Exposure groups

We split the primary analytic sample of adult patients into four groups. Two were considered the groups we expected were most likely to be impacted by immigration-related events and two were considered groups we expected would be less impacted or not impacted. The first group comprised all patients for whom we observed at least one HSF claim in every healthcare encounter throughout the sample period. We call this the “HSF Always” group. After the passage of the Affordable Care Act, most US-born low-income patients became eligible for Medi-Cal and were no longer in the HSF program. Our assumption was that that HSF membership served as a proxy for immigrants who are undocumented or hold other immigration statuses that have been targeted by recent anti-immigration policies and enforcement events. Since the Affordable Care Act, HSF reported that immigration status is the driving factor for why patients are enrolled in HSF instead of other health insurance programs (HSF 2016-2017 Report). The proportion of Latinx enrollees in the program increased rapidly from 27% in FY 2012-2013 (HSF 2013-2014 Report) to nearly 75% in 2014-2015, where it has remained stable for the past few years of reported data (HSF 2014-2015 Report).

The second group we expected would be most impacted includes all Latinx patients who claimed HSF in at least one encounter but were not a part of the HSF Always group. The reason we relax the restriction that the patient must use HSF at every encounter is that healthcare providers attempt to exhaust Medi-Cal resources whenever possible prior to billing HSF.[3] It is thus possible that an HSF member who had even one encounter that was covered by Medi-Cal, such as perinatal care, rather than HSF would not appear in the HSF Always group.

The first group we expected would be less impacted included Latinx patients for whom all observed encounters were billed to Medi-Cal (Medi-Cal Always, Latinx). Our assumption was that those with all encounters billed to Medi-Cal were on unrestricted or regular Medi-Cal, and that this was a proxy for low-income Latinx patients who were U.S. citizens, legal permanent residents, or otherwise held immigration statuses that made them eligible for unrestricted/regular Medi-Cal. Similarly, the second group we expected would be less or not at all impacted is made of up of non-Latinx patients for whom all observed encounters were billed to Medi-Cal (Medi-Cal Always, non-Latinx). Groups were designed to be mutually exclusive; those in the less impacted groups did not include patients who were not in any of the two most impacted groups.

---

[3] For example, prenatal care and labor & delivery are covered by (Emergency) Medi-Cal regardless of immigration status and would be billed to Medi-Cal.

© 2020 Yasenov VI et al. *JAMA Network Open.*

When conducting ancillary analysis for pediatric patients, we had to use other specifications for the comparison groups. There is no way via the electronic medical records to approximate nativity or immigration status of pediatric patients or their parents. As of May 2016, all children in California were eligible for Medi-Cal regardless of immigration status meaning that payer type would therefore not serve as a good proxy for the immigration status of children. We compared utilization outcomes for Latinx compared to non-Latinx children. This mirrors other research focusing on widespread impacts of the 2016 Presidential election and immigration enforcement on Latinx families and children in the US (e.g., Eskenazi et al. 2019, Gemmil et al. 2019, Krieger et al. 2017).

2. Statistical Analysis

We used a difference-in-difference design in a linear regression framework to measure the effect of immigration enforcement events, policies, and related political events on healthcare utilization among probable immigrant patients in San Francisco. We aggregated all outcomes (visits) to the week-group level and then took the log of the outcome. The equation we estimated is:

$$y_{gt} = \alpha + \gamma PostEvent_t * ImpactedGroup_g + \delta_g + \lambda_t + \epsilon_{gt},$$

where g indexes groups and t denotes weeks. The term $PostEvent_t$ is an indicator equal to one for an observation after the event and zero otherwise while $ImpactedGroup_g$ is an impacted group dummy. The coefficient of interest is γ, which measures the differential response in healthcare utilization among the groups we expected would be most impacted relative to the groups we expected would be less impacted in the weeks following each event relative to the weeks leading up to it. Next, δ_g represent group fixed effects which control for permanent differences in healthcare utilization between the four groups. Similarly, λ_t are week fixed effects which account for aggregate trends in hospital visits common to all groups (e.g., lower utilization around major holidays such as Christmas and July 4th). We estimate all regressions on a 10-week window around each of the events described below (5 weeks before and 5 weeks after). To gain statistical precision, we also estimated a specification in which we pooled all 6 events together.

3. Immigration-Related Enforcement, Policy, and Political Events

The events analyzed in this study included political events, policy changes, and rumored or actual immigration enforcement events covering the period from November 2015 to March 2018. One set of events included political and policy changes at the federal level, such as the 2016 US Presidential election, the 2017 Presidential Inauguration, and subsequent passage of anti-immigration Executive Orders.

We also considered local-level immigration enforcement events during this period that occurred in or adjacent to San Francisco County. We included rumored events in additional to actual events, given our expectation that rumors, or anticipated immigration enforcement may have also had an adverse impact on health care utilization (e.g. causing reductions or delays due to fear of traveling or presenting to clinical settings). To identify local-level enforcement events, we used Google to search local newspaper records in the Bay Area for immigration-related events that had widespread media coverage. We additionally evaluated the dates of spikes in pre-identified Spanish and

© 2020 Yasenov VI et al. *JAMA Network Open.*

English-language search terms in the San Francisco Metropolitan Area related to immigration enforcement as defined by Google Trends time-series data.

- Event #1 (Jan 6, 2016) corresponds to the spread of rumored Immigration and Customs Enforcement (ICE) raids in an adjacent county (Oakland and Hayward, CA).[4] This event was also characterized by a spike in Google Trends in enforcement-related terms in the San Francisco Metro Area.

- Event #2 (Nov 8, 2016) was the 2016 US Presidential election. While there were no documented enforcement events on this day, the Trump campaign was characterized by promises to enact anti-immigrant policies, including mass deportation, and to roll-back inclusive policies such as the Deferred Action for Childhood Arrivals (DACA) program.[5]

- Event #3 (Jan 20 – Feb 16, 2017) included the day of and three weeks following the 2017 US Presidential Inauguration. In the aftermath of the Inauguration, the Trump Administration passed a series of anti-immigrant policies such as Executive Order #13769 which, among other things, halted the refugee admission program and enacted a ban against travelers from some Muslim-majority countries,[6] an Executive Order to re-establish participation with local law enforcement agencies to carry out interior immigration enforcement,[7] and Executive Order #13767 which laid the framework to build a wall at the US Southern border.[8] During this same period there was an ICE Raid at Good Samaritan Family Resource Center in San Francisco, CA in which one person was detained. This led to widespread rumors of ICE activity in East and South Bay Area. Given the close temporal proximity of these events, we were not able to distinguish the unique impact of any single one (e.g. the Inauguration only). For multi-week events like this one, we used the earliest date to denote the onset of the event.

- Event #4 (Jul 27–Aug 16, 2017) comprised ICE raids in adjacent cities (Hayward and Oakland, CA) in which 2 detainees were arrested. Although ICE claimed that they were targeting specific individuals with criminal backgrounds, individuals who were not initially targeted were arrested.[9]

---

[4] https://splinternews.com/social-media-is-driving-a-frenzy-of-false-immigration-r-1793853934
https://missionlocal.org/2016/01/sf-advocates-hold-protest-against-recent-immigration-raids/
http://www.sfusd.edu/en/news/current-news/2016-news-archive/01/4751.html
[5] https://www.nytimes.com/2016/09/02/us/politics/transcript-trump-immigration-speech.html
[6] https://tinyurl.com/y9x4xubl
https://www.cnn.com/2017/01/25/politics/donald-trump-immigration-executive-orders/index.html
https://tinyurl.com/yd9e7juf
http://sfist.com/2017/01/26/ice_agents_descend_on_missions_good.php
https://tinyurl.com/y9egdxad
https://sanfrancisco.cbslocal.com/2017/02/16/immigration-and-customs-enforcement-rumors-east-bay-panic/
https://www.nbcbayarea.com/news/local/False-Report-of-ICE-Raid-Causes-Panic-in-East-San-Jose-413905663.html
[7] https://www.whitehouse.gov/presidential-actions/executive-order-enhancing-public-safety-interior-united-states/
[8] https://www.whitehouse.gov/presidential-actions/executive-order-border-security-immigration-enforcement-improvements/
[9] https://tinyurl.com/ybfm7596
https://www.mercurynews.com/2017/07/29/ice-shows-up-to-apartment-complex-looking-for-undocumented-hayward-man-arrests-two-others-instead/
https://www.mercurynews.com/2017/08/06/hayward-officials-raise-questions-about-ice-arrests-of-2-residents/

© 2020 Yasenov VI et al. *JAMA Network Open.*

- Event #5 (Sep 25-29, 2017) comprised widespread ICE raids which took place throughout the San Francisco Bay Area which resulted in 27 detained individuals.[10] Six of these people were detained in San Francisco, CA and the rest in Santa Clara county. These enforcement efforts were part of a nationwide surge in ICE activity targeting undocumented immigrants.[11]

- Event #6 (Jan 10-Feb 1, 2018) comprised another large-scale ICE raid throughout the SF Bay Area.[12]

In the analyses we compared healthcare utilization for comparison groups in the 5 weeks leading up to and following each of these six events. The results when comparing each of the comparison groups separately (e.g., HSF Always vs Medi-Cal Always, Hispanic) were qualitatively very similar, but less precisely estimated. We additionally pooled estimates across all of the events.

4. Multiple Hypothesis Correction

To account for this multiple hypothesis testing, we implemented two corrections that were not pre-registered - the Benjamini-Hochberg (controlling the false discovery rate, FDR) and the Bonferroni-Holm (controlling the family-wise error rate, FWER) procedures.

---

https://tinyurl.com/yalw43lg
https://abc7news.com/society/ice-raid-in-west-oakland-related-to-human-trafficking/2315669/
https://www.nbcbayarea.com/news/local/ICE-HSI-Serve-Federal-Warrant-in-West-Oakland-440781753.html

[10] https://tinyurl.com/ybqdbxjr
https://www.sfgate.com/nation/article/ICE-sweep-targeting-sanctuary-cities-snares-27-in-12239739.php
http://www.sfweekly.com/topstories/27-arrested-by-ice-in-santa-clara-s-f/
https://www.mercurynews.com/2017/09/29/ice-arrests-in-sf-santa-clara-county-targeted-immigrants-with-previous-convictions/

[11] https://tinyurl.com/ybqdbxjr
https://www.sfgate.com/nation/article/ICE-sweep-targeting-sanctuary-cities-snares-27-in-12239739.php
http://www.sfweekly.com/topstories/27-arrested-by-ice-in-santa-clara-s-f/
https://www.mercurynews.com/2017/09/29/ice-arrests-in-sf-santa-clara-county-targeted-immigrants-with-previous-convictions/

[12] https://tinyurl.com/yd2hrb9s
https://www.sfgate.com/bayarea/article/ICE-targets-6-Bay-Area-cities-in-7-Eleven-12488821.php
https://abc7news.com/politics/south-bay-supervisor-calls-ice-raids-at-7-eleven-stores-tragic/2926682/
http://www.ktvu.com/news/ice-plans-major-sweep-in-northern-california-bay-area-report
https://www.mercurynews.com/2018/01/17/it-might-get-ugly-bay-area-cities-brace-for-ice-sweeps/
https://tinyurl.com/y9z4y5w6
https://www.sfgate.com/bayarea/article/ICE-workplace-sweep-hits-Northern-California-12544863.php
https://www.eastbaytimes.com/2018/02/01/ice-targets-77-northern-california-businesses-in-crackdown-on-illegal-workers/

© 2020 Yasenov VI et al. *JAMA Network Open.*

eReferences

Eggli, Y., Desquins, B., Seker, E., & Halfon, P. (2014). Comparing potentially avoidable hospitalization rates related to ambulatory care sensitive conditions in Switzerland: the need to refine the definition of health conditions and to adjust for population health status. *BMC health services research*, *14*(1), 25.

Eskenazi B, Fahey C, Kogut K, Gunier RB, Torres JM, Gonzales NA, Holland N, Deardorff J. Association of perceived immigration policy vulnerability with mental and physical health of among US-born Latino adolescents in California. *JAMA Pediatrics*, 2019, ePub ahead of print.

Gemmil A, Catalano R, Karasek D, Alcalá HE, Casey J, Elser H, Torres JM. Increase in preterm births among US Latina women after the 2016 Presidential election. *JAMA Network Open*, 2019; 2(7): e197084

Krieger N, Huynh M, Li W, Waterman PD, Van Wye G. Severe sociopolitical stressors and preterm births in New York City: 1 September 2015 to 31 August 2017. *J Epidemiol Community Health* 2018;72:1147-1152.

Purdy, S., Griffin, T., Salisbury, C., & Sharp, D. (2009). Ambulatory care sensitive conditions: terminology and disease coding need to be more specific to aid policy makers and clinicians. *Public health*, *123*(2), 169-173.

San Francisco Department of Public Health (2016). Health SF: Our Health Access Program. Annual Report to the San Francisco Health Commission (Fiscal Year 2015-2016). http://healthysanfrancisco.org/wp-content/uploads/2015-16%20HSF%20Annual%20Report.pdf

© 2020 Yasenov VI et al. *JAMA Network Open.*
